# Neuritis and vinblastine-induced axonal transport disruption lead to signs of altered dorsal horn excitability

**DOI:** 10.1177/1744806918799581

**Published:** 2018-11-12

**Authors:** Ieva Satkeviciute, Andrew Dilley

**Affiliations:** 1 Department of Neuroscience, Brighton and Sussex Medical School, University of Sussex, UK

**Keywords:** Neuritis, vinblastine, axonal transport disruption, wide dynamic range neurons, c-Fos, substance P

## Abstract

**Background:**

Many patients with neuropathic pain present without signs of nerve injury on routine clinical examination. Some of these patients may have inflamed peripheral nerves (neuritis). In this study, we have examined whether neuritis causes changes within the dorsal horn that may contribute to a central pain mechanism. Comparisons have been made to a model of axonal transport disruption induced using vinblastine, since neuritis disrupts such processes.

**Results:**

At the peak of cutaneous hypersensitivities, recordings from wide dynamic range neurons revealed increases in wind-up following neuritis but not vinblastine treatment. Ongoing activity from these neurons was unchanged. Vinblastine treatment caused a reduction in the responses of wide dynamic range neurons to noxious mechanical stimulation of the receptive field. The response of neurons to innocuous mechanical stimulation was also reduced in wide dynamic range neurons that were at a depth ≥550 µm following vinblastine treatment. An examination of the superficial dorsal horn revealed an increase in c-Fos–positive neurons in both groups following electrical stimulation of the sciatic nerve. The area of dorsal horn expressing substance P was also decreased following vinblastine treatment.

**Conclusion:**

These findings indicate that a minor nerve insult, such as neuritis, can lead to changes within the dorsal horn that are consistent with a central neuropathic pain mechanism.

## Introduction

Patients diagnosed with conditions such as non-specific arm or back pain, complex regional pain syndrome, whiplash-associated disorder and fibromyalgia often lack signs of frank nerve injury on routine clinical examination despite symptoms that are typically neuropathic in origin. Evidence from a model of local nerve trunk inflammation (neuritis) suggests that some of these patients have inflamed nerves. In this model, animals rapidly develop transient tactile- and thermal-evoked cutaneous hypersensitivities in the absence of axonal degeneration or demyelination.^1–3^ Furthermore, through-conducting nociceptor axons become ongoing and respond to direct mechanical stimulation at the inflammatory site,^3–6^ which may underlie spontaneous and movement-evoked radiating pain that are frequently reported in many of these patients. Although neuritis cannot easily be detected on routine clinical examination, magnetic resonance imaging of the upper limb peripheral nerve trunks in patients with whiplash-associated disorder and diffuse chronic pain syndromes have revealed changes that are consistent with such pathology.^7,8^

The diffuse and often widespread nature of symptoms in these patients has led to the suggestion that some symptoms, such as cutaneous hypersensitivities, are centrally mediated through spinal mechanisms.^9^ Despite studies into the properties of primary sensory neurons following neuritis, spinal cord neurons have not been examined. In contrast, a number of studies have examined the excitability of dorsal horn neurons following nerve ligation and transection, as well as following chronic hind paw inflammation, which dissimilar to neuritis, is associated with significant oedema^10^ and inflammation of the axon terminals.^10^ These studies have demonstrated increases in ongoing activity^11–15^ and receptive field size,^15–18^ which are in line with a central pain mechanism. The majority of electrophysiological studies have focused on a subpopulation of dorsal horn neurons known as wide dynamic range (WDR) neurons. These neurons display a frequency-dependent increase in excitability to repetitive noxious input, called wind-up, which shows similarities with central sensitization.^19^ Consistent with such processes, increases in wind-up have been reported in models of chronic hind paw and joint inflammation.^20–22^

In the present study, we have looked for signs of increased excitability in dorsal horn neurons following neuritis. We have specifically focused on WDR neurons in the deep dorsal horn (i.e. lamina V region). We have also examined c-Fos expression in the superficial dorsal horn in response to electrical stimulation of the sciatic nerve, since these neurons were not examined electrophysiologically. Although the precise role of c-Fos in pain pathways is not well understood, it is a recognized marker of injury-induced and noxious neuronal activation,^23^ as well as an indirect indicator of central sensitization.^24^ Accordingly, c-Fos expression in the superficial dorsal horn is increased following nerve injury.^25–27^ In addition, we have assessed substance P levels in the superficial dorsal horn as a measure of altered nociceptor transmission into the spinal cord, since changes have been reported following nerve injury^28–30^ and chronic hind paw inflammation.^31,32^

We have previously shown that neuritis disrupts fast anterograde axonal transport, and have hypothesized that such altered processes are important in the mechanisms that lead to pain.^33^ The role of axonal transport disruption along intact, through conducting axons can be examined without inflammation by applying low doses of the vinca alkaloid, vinblastine, to the sciatic nerve.^34–38^ Following vinblastine treatment, animals develop tactile-evoked cutaneous hypersensitivity and nociceptor axons respond to mechanical stimulation at the treatment site.^33,34,38^ To determine whether axonal transport disruption contributes to central processes, we have compared neuritis to vinblastine treatment. To confirm the optimum time-point for the assessment of dorsal horn neurons, neuropathic pain behaviors were also examined. Together, our findings show that neuritis causes changes in the dorsal horn that may contribute to a central neuropathic pain mechanism.

## Materials and methods

### Animals

Experiments were carried out in strict accordance with the UK Animals (Scientific Procedures) Act (1986) and Home Office guidelines. Adult male Sprague Dawley rats (*n* = 97; 175–500 g; Charles River Laboratories, Kent, UK) were used in this study. Rats were housed in groups of two to four in cages with sawdust and soft nesting material and were given ad libitum access to food and water. Animals were monitored daily for adverse effects.

### Surgery

Neuritis was induced as previously described (*n* = 30 animals).^33^ Under general anesthesia (1.75% isoflurane in oxygen), a short incision parallel to the femur was made through the skin in the mid-thigh on the left side. The underlying muscle was separated by blunt dissection to expose the sciatic nerve, and the connective tissue surrounding an 8-mm length of the nerve was carefully cleared. A length of sterile Gelfoam (Spongostan™; Ferrosan, Denmark), ∼6 × 20 × 2 mm, saturated in 50% complete Freund’s adjuvant (diluted in sterile 0.9% w/v saline) was wrapped loosely around the sciatic nerve, and the muscle and skin were closed with 4/0 monofilament sutures (Vicryl; Ethicon, West London, UK).

Vinblastine (*n* = 27 animals) and saline (*n* = 17 animals) were applied to the sciatic nerve as previously described.^33^ The left sciatic nerve was exposed in the mid-thigh by blunt dissection and the connective tissue surrounding an 8-mm length of the nerve was carefully cleared. A strip of sterile parafilm (Sigma Aldrich, Dorset, UK), ∼6 × 20 mm, was positioned under the nerve to prevent leakage of agents onto the underlying muscle. A length of Gelfoam (∼6 × 20 × 2 mm) saturated in either 0.1 mM vinblastine (diluted 0.9% w/v saline) or 0.9% w/v saline was wrapped loosely around the nerve. After 15 min, both the Gelfoam and parafilm were removed, and the nerve was rinsed with sterile saline. The muscle and skin were closed with sutures.

### Behavior

Behavioral testing was always performed at the same time on each day. Animals were habituated to the test apparatus for 1 h on 3 consecutive days before the start of the testing period. On the day of testing, animals were acclimatized for 15 min to allow exploration and major grooming activities to cease. For each behavior, both hind paws were tested with a 5-min rest period between sides. The side to be tested first was randomized. Six animals were examined in each treatment group. Tactile- and cold-evoked hypersensitivities were tested on different animals.

Tactile-evoked hypersensitivity was assessed using calibrated von Frey monofilaments of increasing stiffness (0.4, 0.7, 1.2, 2, 4, 6, 9, 15 g; Ugo Basile, Varese, Italy) on days 1, 4, 5, 6, 7, 8 and 11 postoperative. Animals were placed into individual acrylic glass enclosures raised on a metal-perforated floor. Von Frey hairs were applied perpendicular to the plantar surface of the glabrous foot and held for 5 s. A positive response was recorded if the paw was sharply withdrawn, held or licked. Paw movements due to postural changes were not considered to be positive responses. The 50% paw withdrawal threshold was determined using the up–down method.^39,40^ Testing commenced with the 2.0 g hair. If no response was initiated, von Frey hairs of increasing stiffness were presented. A negative response, followed by the first positive response, was considered the response threshold. At the response threshold, hairs of decreasing stiffness were applied to the point that there was no response. At this point, von Frey hairs of increasing stiffness were applied until the next positive response was recorded. This pattern was repeated moving up and down von Frey hairs until a maximum of four stimuli had been applied after the response threshold. If all responses were only negative or positive, values of 15.0 g or 0.4 g were assigned respectively. The following formula was used to determine the 50% paw withdrawal threshold: 50% g threshold = (10^[*X*_f_ + *k*δ])/10,000; where *X*_f_ = value of the final von Frey hair used (in log units); *k* = tabular value for the pattern of positive/negative responses^40^; δ = mean difference in log units between stimuli (here, 0.224). There was a 1-min interval between each von Frey hair application.

Cold-evoked hypersensitivity was tested on days 1, 2, 3, 4, 8, 14 and 21 postoperative using the acetone test as previously described.^41^ An acetone bubble (10 µl) was formed at the end of a polyethylene tube that was connected to a syringe. With the animals in individual acrylic glass enclosures, the bubble was carefully applied to the plantar surface of the foot through the perforated floor. Care was taken to ensure that the tubing did not touch the skin. A brisk foot withdrawal was considered a positive response. Acetone was applied five times to each paw at 2.5-min intervals. The percentage paw withdrawal frequency was calculated.

### Electrophysiology

**Figure 1.**
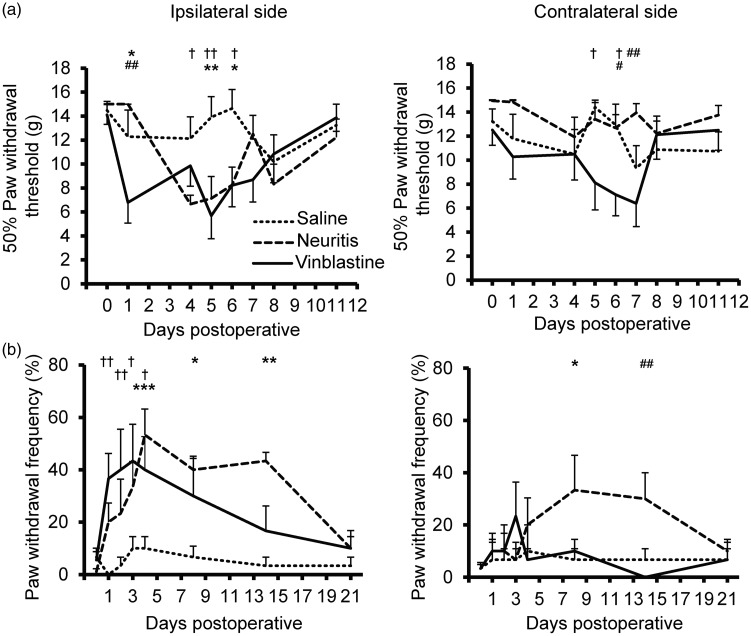
Development of cutaneous hypersensitivities. (a) Tactile- and (b) cold-evoked hypersensitivities in saline-treated, neuritis and vinblastine-treated groups (*n* = 6 animals/group). Ipsilateral (left) and contralateral (right) data are shown for each test. Each data point represents the mean of six animals. *p < 0.05, **p < 0.01, ***p < 0.001 comparing neuritis to saline-treated group; ^†^*p* < 0.05, ^††^*p* < 0.01 comparing vinblastine-treated to saline-treated group; ^#^*p* < 0.05, ^##^*p* < 0.01, comparing neuritis to vinblastine-treated group (two-way ANOVA followed by Bonferroni’s post hoc tests at each time point). Error bars = SEM. SEM: standard error of the mean.

Recordings from dorsal horn WDR neurons in the region of the L5 spinal segment were performed in 11 untreated, 8 saline-treated, 9 neuritis and 6 vinblastine-treated animals. The L5 spinal segment innervates much of the plantar surface of the foot that was examined during behavioral testing. The methods were based on those previously described.^42^ Experiments were carried out 3–6 days postoperative, that is at the approximate peak of pain behaviors (Figure 1). Animals were deeply anesthetized (1.75 g/kg 25% w/v urethane i.p.), and the body temperature maintained at 37°C using a rectal thermistor probe attached to a feedback controlled heat mat (Harvard Apparatus, Kent, UK). A mid-sagittal skin incision was made in the lumbar region and a short laminectomy (L1–L3 vertebrae) was performed to expose the L5 spinal cord segments (i.e. the lumbosacral enlargement). The body of the rat was secured by hip pins and the skin flaps were sutured to a metal ring to form a pool. The dura mater was cut to expose the spinal cord, and the pool was filled with mineral oil warmed to 37°C. The vertebral column was fixed in place using clamps attached to the spinous processes immediately rostral and caudal of the exposed spinal cord. An insulated tungsten electrode (76 mm, 2.0 MΩ, 1 µm tip, 0.127 mm shaft diameter; World Precision Instruments, Sarasota, FL, USA) was lowered onto the pia surface of the left side of the spinal cord, immediately lateral to the posterior spinal artery, using a micro-manipulator. A reference electrode was attached to the metal ring.

**Figure 2.**
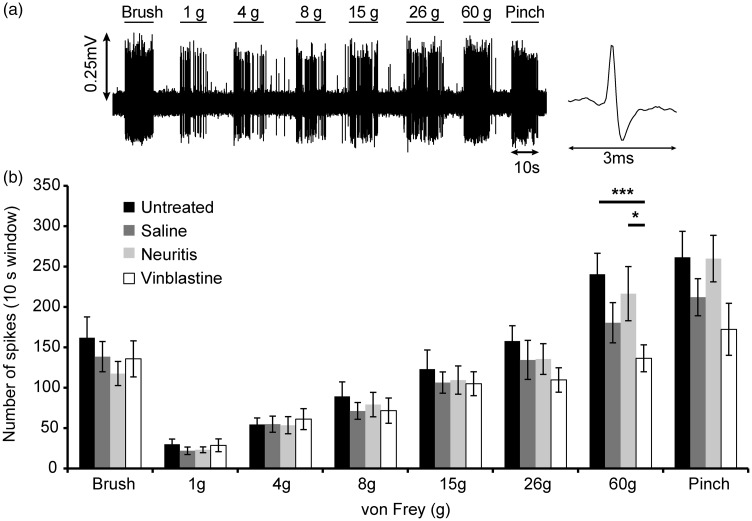
Responses of WDR neurons to mechanical stimulation of the receptive field. (a) An example trace from a saline-treated animal, which shows action potentials evoked by lightly brushing the receptive field, applying von Frey hairs of ascending stiffness and pinching the skin with curved forceps. (b) The mean number of action potentials elicited by brushing, von Frey monofilaments and pinching the skin in untreated, saline-treated, neuritis and vinblastine-treated animals. Note the decreased response to stiffer von Frey monofilaments and pinching in the vinblastine-treated group. *n* = 19, 18, 20 and 13 WDR neurons in the untreated, saline-treated, neuritis and vinblastine-treated group respectively. *p < 0.05, ***p < 0.001 (two-way ANOVA followed by Bonferroni’s post hoc tests). Error bars = SEM.SEM: standard error of the mean.

The tungsten electrode was slowly lowered into the dorsal horn in 10- to 50-µm steps to a depth of 200–900 µm while the ipsilateral foot was stroked and/or gently tapped. Based on a histological examination of the spinal cord, this depth was considered the lamina V region (data not presented). Once a response could be recorded (i.e. action potentials evoked by innocuous brushing of the skin), the center of the receptive field was identified, and the neuron was monitored for 3 min to assess ongoing activity levels. Neurons that fired at least once per min (0.017 Hz) were considered ongoing. The neuron was then characterized by assessing its responses to a range of mechanical stimuli applied to the receptive field. Initially, the center of the receptive field was lightly brushed, followed by the application of an ascending series of von Frey monofilaments (1, 4, 8, 15, 26 and 60 g), after which the receptive field was squeezed using curved forceps. Each stimulus was applied for 10 s, with a 10-s interval between stimuli. A neuron was considered a WDR neuron if there was a clear graded response to the applied stimuli (Figure 2). For assessment of wind-up, two needle electrodes were inserted transcutaneously into the center of the receptive field and 16 consecutive electrical stimuli (0.5 Hz, 2 ms pulse width) were applied at 3× the C-fiber threshold (0.75–5 V). The number of action potentials that were evoked by Aβ-, Aδ- and C-fiber neuron input was determined. Action potentials with latencies between 0–20 ms, 20–90 ms and 90–350 ms were considered to be evoked by Aβ-, Aδ- and C-fiber neurons, respectively. Action potentials with latencies between 350 and 800 ms were classified as post-discharge. Wind-up was calculated from the sum of the action potentials between 90 and 800 ms evoked by the 16 consecutive stimuli (i.e. total C-fiber–evoked responses and post-discharge) minus the input, which was calculated from the sum of the C-fiber–evoked response and post-discharge after the first electrical stimulus multiplied by 16. Neurons with positive values were considered to show signs of wind-up. Action potentials were amplified (1 K), band-pass filtered (10–5000 Hz) and monitored with an oscilloscope. Neuronal activity was digitized and recorded with Spike 2 software (Cambridge Electronic Designs, Cambridge, UK) for offline analysis. The total number of neurons examined in each animal ranged from 1 to 5. To avoid possible sensitization of the skin due to damage, only neurons with non-overlapping receptive fields were examined in the same animals.

### Immunohistochemistry

The expression of c-Fos and substance P in the spinal cord was measured by immunohistochemistry. All animals were examined on day 4–5 postoperative. For the examination of electrically evoked c-Fos expression, 12 untreated, 12 neuritis and 12 vinblastine-treated animals were deeply anesthetized using pentobarbital (50 mg/kg i.p.). In four animals from each of the treatment groups, the ipsilateral sciatic nerve was exposed in the mid-thigh and stimulated for 5 min at A-fiber strength (3 V, 0.05 ms, 5 Hz) using bipolar electrodes positioned under the nerve. In another four animals from each group, the nerve was stimulated at C-fiber strength (30 V, 0.5 ms, 5 Hz). The voltage necessary to stimulate A- and C-fiber neurons was determined during preliminary in vivo electrophysiology experiments (data not presented). After 1 h, all animals were transcardially perfused with ice-cold 0.1 M phosphate-buffered saline (PBS) followed by ice-cold 4% paraformaldehyde (in 0.1 M PBS, pH 7.4). For examination of substance P, three saline-treated, three neuritis and three vinblastine-treated animals were anesthetized using pentobarbital (50 mg/kg i.p.) and perfused as described above. Note that for c-Fos examination, untreated rather than saline-treated animals were examined. Behavioral and electrophysiological assessment of animals confirmed that there was negligible difference between saline-treated and untreated groups at the time-point examined.

The L5 spinal cord segment was removed and post-fixed in 4% paraformaldehyde for 60 min and then transferred to 30% sucrose (in 0.1 M PBS) overnight at 4°C for tissue cryoprotection. Spinal cords were snap frozen in isopentane on dry ice and transverse sections were cut at 20 µm using a cryostat (Leica Microsystems, Wetzlar, Germany) and thaw-mounted on gelatin-coated glass slides. For c-Fos examination, approximately three sections from an untreated, neuritis and vinblastine-treated animal that underwent similar electrical stimulation were mounted on the same slide. For substance P examination, approximately three sections from a saline-treated, neuritis and vinblastine-treated animal were mounted on the same slide. Two slides were examined for each animal.

Sections were blocked in 3% normal goat serum (Vector Laboratories, Burlingame, USA) in 0.2% Triton X-100 for 1 h at room temperature and incubated overnight at 4°C with c-Fos polyclonal rabbit (1:1000; sc-52; Santa Cruz Biotechnology, Dallas, TX, USA) or substance P monoclonal mouse (1:1000; ab14184; Abcam, Cambridge, UK) primary antibodies. The sections were then incubated for 1 h at room temperature with Alexa Fluor 488 goat anti-rabbit (1:200; Thermo Fisher Scientific, Paisley, UK) or Alexa Fluor 546 goat anti-mouse (1:500; Thermo Fisher Scientific) conjugated secondary antibodies respectively, and coverslipped using glycerol/PBS mounting medium (Citiflour, London, UK). All antibodies were diluted in 3% normal goat serum and 0.2% Triton X-100. Between each step, slides were washed in PBS (3 × 5 min). For double-labeling experiments, c-Fos polyclonal rabbit (1:1000) and NeuN monoclonal mouse (1:100; MAB377; Millipore Corporation, Bedford, MA, USA) primary antibodies were incubated together followed by Alexa Fluor 488 goat anti-rabbit (1:200; Thermo Fisher Scientific) and Alexa Fluor 546 goat anti-mouse (1:200; Thermo Fisher Scientific) conjugated secondary antibodies using the same protocols.

Slides were viewed under a fluorescence microscope (Leica Microsystems) at 488 and 546 nm excitation and photographed at 100× at each wavelength using the same settings. In addition, double-labeled sections were viewed under a confocal microscope (Leica Microsystems SP8) at 488- and 546-nm excitation and photographed at 200× at each wavelength. Non-specific staining with secondary antibody was not found in the absence of primary antibody in any case.

### Data analysis

Data were tested for normality using Shapiro–Wilk tests. For behavioral data, comparisons between groups at each time-point were performed using two-way analysis of variance (ANOVA) followed by Bonferroni’s post hoc tests. For electrophysiological data, comparisons between groups were performed using one-way (brushing receptive field, pinching receptive field, electrical stimulation of receptive field and wind-up responses) or two-way ANOVAs (von Frey monofilament responses) followed by Bonferroni’s post hoc tests. The proportion of ongoing activity was compared using Fisher’s exact tests, and rates were compared using Kruskal–Wallis tests. Animal weights were compared using Student’s *t* tests. For analysis of c-Fos labeling, images of the dorsal horn were converted to greyscale using ImageJ software (US National Institutes of Health, Bethesda, MD, USA). A region of interest was drawn around the superficial dorsal horn as follows: a line was drawn from medial to lateral following the posterior surface of the dorsal horn. At the lateral margin, the line was extended anteriorly around the curve of the dorsal horn, and a straight line was drawn parallel to the posterior surface, medially, to reach the dorsal column. This line was then extended to the posterior surface (see insets on Figure 5). The area of the region of interest was measured. The threshold was set so that individual cells could be identified. From the resulting binary image, the number of positively labeled cells within the region of interest was determined using the “analyze particles” function within ImageJ software. To allow for variations in the size of the region of interest, the number of positive cells per 0.1 mm^2^ was calculated for each section, and estimates from multiple sections of the same dorsal horn (*n* = 3 sections from each animal) were averaged. Numbers of positive cells per 0.1 mm^2^ were compared between treatment groups and sides using two-way ANOVAs followed by Bonferroni’s post hoc tests. For analysis of substance P labeling, images of the dorsal horn were converted to greyscale using ImageJ software. For each image, a greyscale value that was two standard deviations above the mean was determined from an area approximately 140 × 75 pixels at the base of the dorsal horn. This value was used for thresholding the image. The area of positive labeling (i.e. above the threshold) within the superficial lamina was measured. A ratio for the difference in the size of the area expressing substance P on the ipsilateral compared to contralateral side was calculated. A value <1 indicated the area with positive labeling was less on the ipsilateral compared to contralateral side; a value >1 indicated the area with positive labeling was greater on the ipsilateral compared to contralateral side. Mean area ratios were compared using a one-way ANOVA followed by Bonferroni’s post hoc tests. Data are presented as mean ± standard error of the mean. The ongoing activity rates are presented as median ± interquartile range.

## Results

### Development of cutaneous hypersensitivities

Both neuritis and vinblastine treatment caused reversible signs of tactile-evoked cutaneous hypersensitivity (Figure 1(a)). On the ipsilateral side, there was a significant interaction between treatment groups and von Frey threshold at each time point (*F*(14, 112) = 2.25, *p* < 0.01, two-way ANOVA; *n* = 6 per group; Figure 1(a)). Following vinblastine treatment, a reduction in threshold was evident as early as day 1 postoperative. A maximum reduction in von Frey threshold was observed on days 4–5 in both neuritis and vinblastine-treated groups (*p* < 0.01 compared to saline treatment, Bonferroni’s post hoc test). A reversal was observed by day 11 in both groups. On the contralateral side, there was no significant interaction (*F*(14, 112) = 1.21, *p* = 0.28). However, there was a significant main effect for group (*F*(2, 112) = 9.84, *p* < 0.001, two-way ANOVA), with signs of tactile hypersensitivity following vinblastine treatment. Note the duration of the hypersensitivity was shorter compared to the ipsilateral side (Figure 1(a)).

Both neuritis and vinblastine treatment caused reversible signs of cold-evoked cutaneous hypersensitivity (Figure 1(b)). On the ipsilateral side, there was a significant interaction between treatment groups and paw withdrawal frequency at each time point (*F*(14, 105) = 2.86, *p* < 0.01, two-way ANOVA; Figure 1(b)). Following vinblastine treatment, an increase in paw withdrawal frequency was observed as early as day 1 postoperative. A maximum paw withdrawal frequency occurred on days 3–4 in both neuritis and vinblastine-treated groups (*p* < 0.05 compared to saline treatment, Bonferroni’s post hoc test). A reversal was observed by days 14 and 21 following vinblastine-treatment and neuritis, respectively. On the contralateral side, there was also a significant interaction (*F*(14, 105) = 1.94, *p* < 0.05), with signs of cold hypersensitivity in the neuritis group (Figure 1(b)).

### Electrophysiological properties of wide dynamic range neurons

Recordings were made from 70 dorsal horn WDR neurons in untreated (*n* = 19 neurons), saline-treated (*n* = 18 neurons), neuritis (*n* = 20 neurons) and vinblastine-treated (*n* = 13 neurons) animals. The neurons were located at a mean depth of 551 µm (SD = 143; range: 200–900 µm) from the dorsal surface of the spinal cord. Following isolation of each WDR neuron, background ongoing activity was recorded. The proportion of neurons with ongoing activity was similar between groups, with > 75.0% of neurons firing spontaneously (i.e. > 0.017 Hz; *p* > 0.18, Fisher’s exact tests; Table 1). The median rates of ongoing activity were slow, ranging from 0.06–0.24 Hz, with no significant difference between groups (*p* = 0.32, Kruskal–Wallis test). The pattern of firing was irregular.

**Table 1.** Levels (%) and rates (Hz) of ongoing activity in WDR neurons.

Treatment	Number of WDR neurons	%OA	Rate, median (IQR)
Untreated	19	89.5	0.12 (0.13)
Saline	18	94.4	0.13 (0.19)
Neuritis	20	75.0	0.06 (0.20)
Vinblastine	13	76.9	0.24 (0.08)

OA: ongoing activity; IQR: interquartile range; WDR: wide dynamic range.

For all WDR neurons, action potentials were evoked in response to both innocuous and noxious mechanical stimulation of the ipsilateral distal hind paw (Figure 2(a) and (b)). The number of action potentials evoked by brushing the center of each receptive field was comparable between groups (*F*(3, 66) = 0.86, *p* = 0.47, one-way ANOVA). There was a significant interaction between treatment group and von Frey monofilament stiffness (*F*(15, 330) = 1.94, *p* < 0.05, two-way ANOVA). In the untreated, saline-treated and neuritis groups, mechanical stimulation of the receptive field with increasing force caused a progressive increase in the number of evoked action potentials. In the vinblastine-treated group, the response plateaued with stiffer von Frey monofilaments. The number of action potentials evoked with the 60 g von Frey monofilament was significantly less following vinblastine treatment compared to the untreated (*p* < 0.001) and neuritis groups (*p* < 0.05, Bonferroni’s post hoc tests). The number of evoked action potentials in response to pinching the skin was comparable between groups (*F*(3, 66) = 1.90, *p* = 0.14, one-way ANOVA; Figure 2(b)).

**Figure 3.**
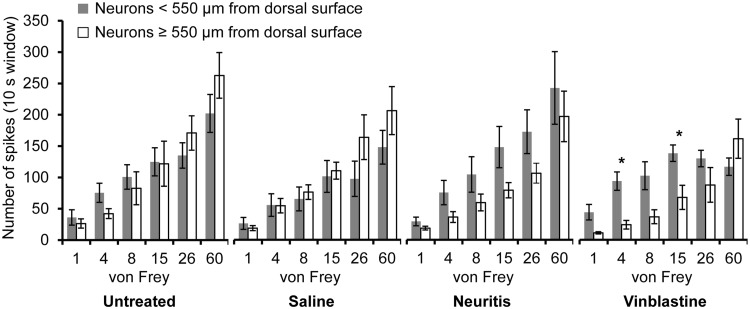
Mechanical responses of WDR neurons based on depth of neuron within the dorsal horn. *n* = 7, 8, 9 and 7 <550 µm and 12, 10, 11 and 6 ≥550 µm from dorsal surface of spinal cord in the untreated, saline-treated, neuritis and vinblastine-treated groups respectively. *p < 0.05 (two-way ANOVA followed by Bonferroni’s post hoc tests).

To assess the effect of neuronal depth on the response to mechanical stimulation of the receptive field, WDR neurons were grouped into those ≥550 µm (i.e. the mean depth of all neurons; maximum depth = 900 µm) from the dorsal surface of spinal cord and those <550 µm (minimum depth = 200 µm). In the untreated and saline-treated groups, there was no significant interaction between responses evoked by each von Frey filament and neuronal depth (untreated: *F*(5, 85) = 2.22, *p* = 0.06; saline-treated: *F*(5, 80) = 2.00, *p* = 0.09) or main effect for depth (untreated: *F*(1, 17) = 0.03, *p* = 0.87; saline-treated: *F*(1, 16) = 0.80, *p* > 0.39, two-way ANOVA). In both the neuritis and vinblastine-treated groups, neurons ≥550 µm from the dorsal surface of the spinal cord responded less to lower force von Frey monofilaments (1–15 g) compared to those <550 µm from the surface. In the vinblastine-treated group, there was a significant interaction between neuronal depth and von Frey monofilament stiffness (*F*(5, 55) = 6.09, *p* < 0.001, two-way ANOVA). In this group, there was a plateauing in responses from neurons <550 µm from the dorsal surface of the spinal cord to 8- to 60-g von Frey monofilaments that was not observed from deeper neurons (Figure 3). In the neuritis group, there was no significant interaction (*F*(5, 90) = 0.65, *p* = 0.66) or main effect for depth (*F*(1, 18) = 2.53, *p* = 0.13, two-way ANOVA). As the depth of neuron is also related to the size of the animal, animal weights were compared between experiments that examined neurons ≥550 µm and <550 µm from the surface of the spinal cord. There was no significant difference between animal weights in the saline-treated, neuritis or vinblastine-treated groups, although there was a difference in the untreated group (Table 2).

**Table 2.** Comparison of animal weight in experiments that examined WDR neurons <550 µm and ≥550 µm from the dorsal surface of the spinal cord.

	Animal weight, mean (SEM)	
Treatment	Neurons<550 µm	Neurons≥550 µm
Untreated	292 (18)	373 (20)*
Saline	322 (18)	353 (20)
Neuritis	388 (17)	390 (20)
Vinblastine	329 (5)	325 (17)

SEM: standard error of the mean; WDR: wide dynamic range.

* *p* < 0.05 (Student’s *t* test).

**Figure 4.**
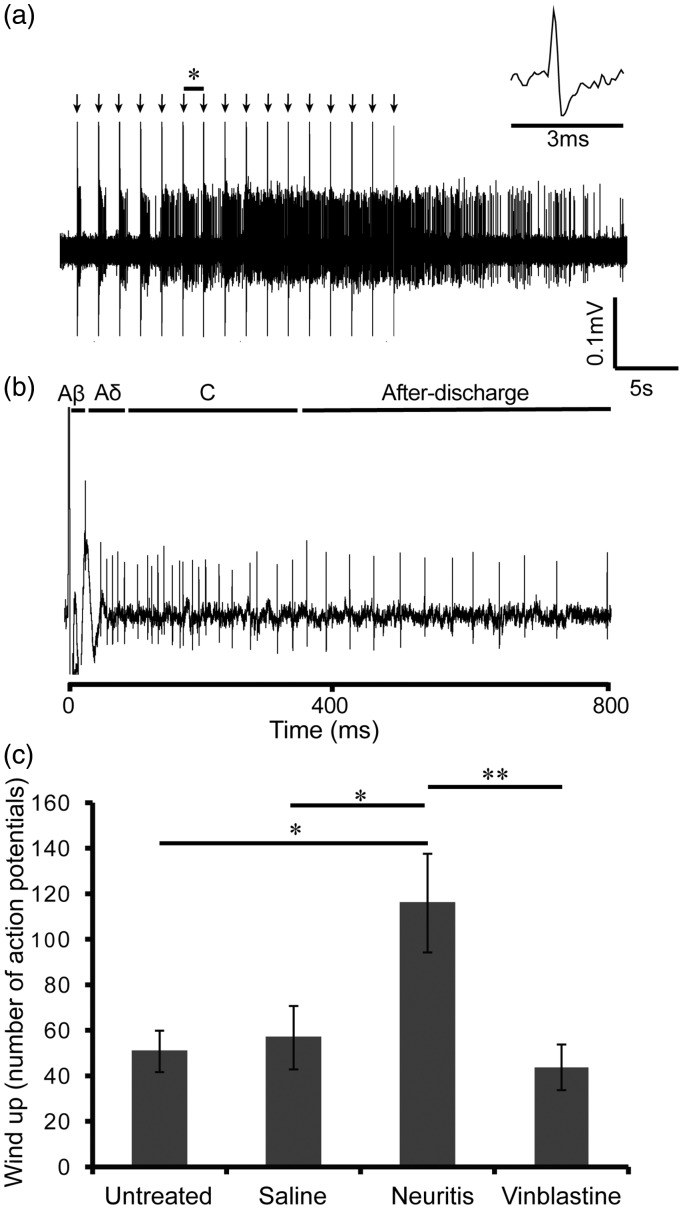
Wind-up responses. (a) Typical wind-up response from a neuritis animal. The center of the receptive field was electrically stimulated 16 times at 3× C-fiber threshold (arrows). (b) Expanded section of trace (* on part (a)), showing action potentials evoked by electrical stimulation. Action potentials were classified as Aβ-, Aδ-, or C-fiber–evoked based on the following latencies: Aβ, 0–20 ms; Aδ, 20–90 ms; C, 90–350 ms. Action potentials that occurred at a latency of 350–800 ms were considered post-discharge. (c) A comparison of the mean wind-up responses of WDR neurons in untreated, saline-treated, neuritis and vinblastine-treated groups. Wind-up was calculated as the sum of action potentials between 90 and 800 ms produced by 16 consecutive stimuli minus the input. *n* = 10, 11, 11 and 10 WDR neurons in the untreated, saline-treated, neuritis and vinblastine-treated groups, respectively. *p < 0.05, **p < 0.01 (one-way ANOVA followed by Bonferroni’s post hoc tests). Error bars = SEM.SEM: standard error of the mean.

In total, 42 of the characterized WDR neurons showed signs of wind-up in response to repeated electrical stimulation (16×) of the receptive fields (untreated group, *n* = 10 neurons; saline group, *n* = 11 neurons; neuritis group, *n* = 11 neurons; vinblastine group, *n* = 10 neurons; e.g. Figure 4(a)). The total number of Aβ-, Aδ- and C-fiber–evoked responses (e.g. Figure 4(b)), input (i.e. number of action potentials evoked by first pulse × total number of pulses) and post-discharge of these neurons are summarized in Table 3. There were no significant differences between groups in the number of action potentials evoked by Aβ-fiber neurons (*F*(3, 37) = 0.85, *p* = 0.47, one-way ANOVA). Although not quite significant, there was a notable reduction in the number of action potentials evoked by Aδ- and C-fiber neurons in the vinblastine-treated group compared to neuritis (Aδ-fiber neurons: *F*(3, 37) = 3.17, *p* < 0.05; C-fiber neurons: *F*(3, 37) = 2.97, *p* < 0.05, one-way ANOVA; *p* = 0.06 and 0.07, respectively, Bonferroni’s post hoc tests). Both input and post-discharge were comparable between groups (input: *F* (3, 66) = 1.25, *p* = 0.30; post-discharge: *F*(3, 66) = 1.59, *p* = 0.20, one-way ANOVA).

**Table 3.** Comparison of the mean total electrically evoked responses of WDR neurons in untreated, saline-treated, neuritis and vinblastine-treated groups.

	Treatment group			
	Untreated	Saline	Neuritis	Vinblastine
Aβ-fiber	7.90 (3.57)	16.64 (8.09)	12.30 (6.47)	4.60 (2.00)
Aδ-fiber	29.10 (6.92)	46.09 (11.84)	50.00 (8.88)	15.60 (5.87)
C-fiber	209.90 (27.68)	158.36 (20.52)	229.70 (35.27)	129.40 (21.18)
Input	233.60 (31.21)	162.91 (31.47)	224.00 (46.86)	132.80 (33.23)
Post-discharge	74.40 (12.85)	61.27 (16.98)	110.10 (24.47)	47.10 (15.31)

Data are expressed as mean number of spikes (SEM). SEM: standard error of the mean; WDR: wide dynamic range.

The wind-up response was similar between the untreated (mean wind-up = 50.70 ± 9.07 action potentials), saline- (mean wind-up = 56.73 ± 13.89 action potentials) and vinblastine-treated groups (mean wind-up = 43.70 ± 10.05 action potentials). However, in the neuritis group there was a significant increase in wind-up response (mean = 115.80 ± 21.67 action potentials, *F* (3, 37) = 5.10, *p* < 0.01 one-way ANOVA; *p* < 0.05 compared to untreated, saline-treated and vinblastine-treated groups, Bonferroni’s post hoc tests; Figure 4(c)).

### c-Fos immunolabeling

**Figure 5.**
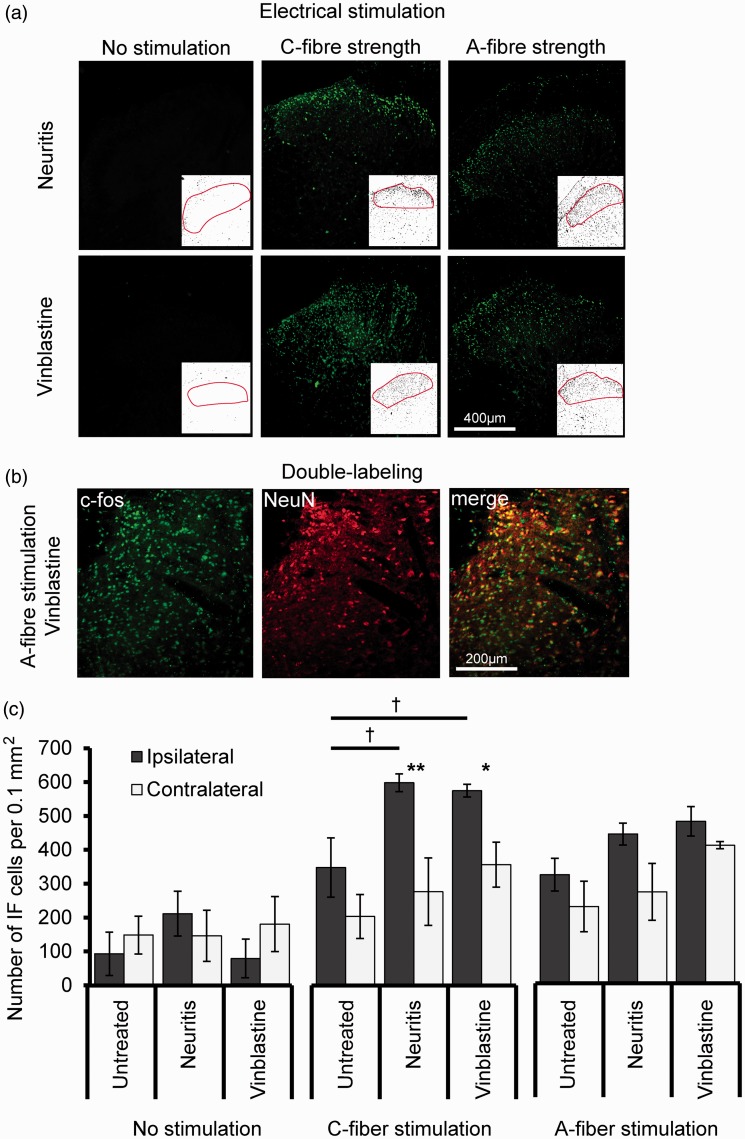
Electrical stimulation-activated c-Fos expression in the L5 dorsal horn. (a) Example images of c-Fos expression (green staining) in the ipsilateral dorsal horn following neuritis and vinblastine treatment. Prior to removal of the tissue, animals received either 5-min repeated stimulation of the sciatic nerve at C- (middle) or A-fiber strength (right), or no stimulation (left). The majority of c-Fos positive cells were located in the superficial laminae. Note inserts that represent the respective image thresholding used for ImageJ analysis. (b) Colocalization of c-Fos (green) with NeuN (red). (c) Mean number of c-Fos positive cells per 0.1 mm^2^ in each group (*n* = 4 animals in each group). ^†^*p* < 0.05, comparing between treatments; *p < 0.05, **p < 0.01 comparing between sides (two-way ANOVA followed by Bonferroni’s post hoc tests). Error bars = SEM.IF: Immunofluorescent; SEM: standard error of the mean.

C-Fos was expressed in cells within the superficial lamina of the dorsal horn in all groups (*n* = 4 animals in each group; Figure 5(a)). Double-labeling with neuronal marker NeuN confirmed that the majority of c-Fos–labeled cells were neurons (Figure 5(b)). In the unstimulated groups, there was no significant interaction between treatment and side for the numbers of c-Fos positive cells (*F*(2, 6) = 3.84, *p* = 0.08) or main effect (*F*(1, 3) = 2.34, *p* > 0.23, two-way ANOVA; Figure 5(c)). Repeated electrical stimulation of the sciatic nerve at C-and A-fiber strength caused an increase in c-Fos expression in all groups. In the groups stimulated at C-fiber strength, there was no significant interaction between treatment and side (*F*(2, 6) = 1.67, *p* = 0.27). However, there were significant main effects for treatment (*F*(2, 6) = 6.94, *p* < 0.05) and side (*F*(1, 3) = 113.80, *p* < 0.01, two-way ANOVA), with increased c-Fos expression on the ipsilateral side in the neuritis and vinblastine-treated groups compared to the untreated group and contralateral sides (*p* < 0.05, Bonferroni’s post hoc tests). In the groups stimulated at A-fiber strength, there was no significant interaction between treatment and side (*F*(2, 6) = 1.35, *p* = 0.33) or main effect (*F*(2, 6) = 4.68, *p* = 0.06, two-way ANOVA).

### Substance P immunolabeling

**Figure 6.**
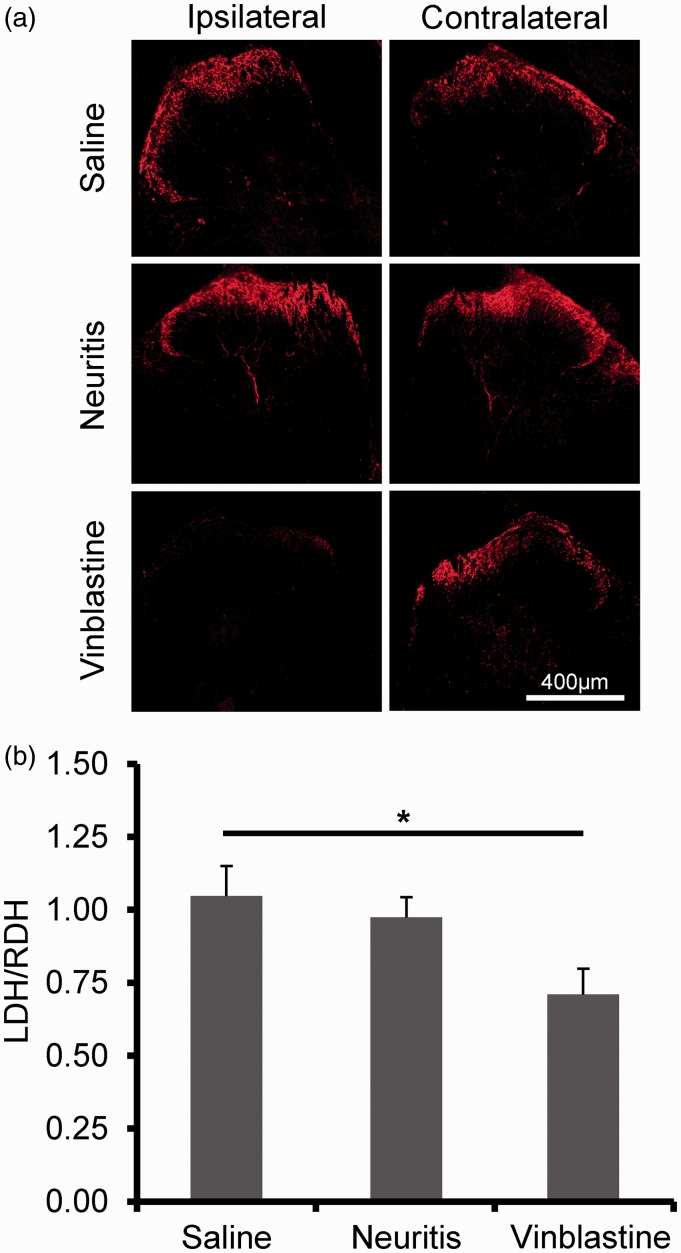
Substance P expression in the dorsal horn. (a) Typical example images of substance P expression in the L5 region of the dorsal horn in saline-treated, neuritis and vinblastine-treated groups. The majority of expression is observed within the superficial lamina (red staining). (b) Mean ratios of the dorsal horn area expressing substance P. Calculation was made by dividing the ipsilateral area by the contralateral area. *n* = 3 animals in each group. *p < 0.05 (one-way ANOVA followed by Bonferroni’s post hoc test). Error bars = SEM.ANOVA: analysis of variance; LDH: Left dorsal horn; RDH: Right dorsal horn; SEM: standard error of the mean.

There was notable substance P immunolabeling in the superficial dorsal horn on both the ipsilateral and contralateral sides following saline-treatment and neuritis (*n* = 3 animals in each group; Figure 6(a)). In these groups, the area of dorsal horn expressing substance P was comparable between sides (mean ratio [ipsilateral area/contralateral area] = 1.05 ± 0.10 in the saline-treated and 0.97 ± 0.07 in the neuritis groups). Following vinblastine treatment, the area of dorsal horn expressing substance P was reduced on ipsilateral compared to contralateral side (mean ratio = 0.71 ± 0.09; *F*(2, 14) = 4.31, *p* < 0.05, one-way ANOVA; *p* < 0.05, Bonferroni’s post hoc test; Figure 6(b)).

## Discussion

In this study, we have shown that, at approximately the peak of neuropathic pain behaviors, localized neuritis leads to an increase in dorsal horn excitability. In contrast, there were no signs of an increase in the excitability of WDR neurons following vinblastine treatment, despite an increase in c-Fos expression in superficial laminae. In both models, cutaneous hypersensitivities and spinal cord changes occur in the absence of significant axonal degeneration or demyelination, which was demonstrated in previous studies.^1,3,33,38^ Taken together, these findings indicate that dorsal horn plasticity can be triggered by a relatively minor insult to a nerve.

The increase in wind-up response from deep WDR neurons following neuritis suggests that this neuronal population is sensitized, which is in line with similar reports of increased wind-up in chronic inflammatory models (e.g. injection of complete Freund’s adjuvant or carrageenan into the hind paw or joints).^20–22^ Consistent with a role for inflammation, the wind-up response did not change following vinblastine treatment, since vinblastine is, by virtue of its anti-mitotic properties, anti-inflammatory.^37^ The increase in wind-up following neuritis contrasts from observations in models of neuropathic pain that involve cutting or constricting nerves, where similar responses are not reported.^11,12,14,43^ These discrepancies may reflect differences in the inflammatory response between models, as well as the pattern of peripheral ongoing activity, which is reputed to drive increases in dorsal horn excitability.^44–47^ For example, ongoing activity is reported from nociceptor neurons following neuritis,^3,6,48^ and in chronic inflammatory models, ^49–52^ but not following vinblastine treatment; in fact, ongoing activity is not increased in any fiber type in this model.^33,53^ In models of neuropathic pain that involve traumatic nerve injury, ongoing activity from nociceptor neurons is rarely observed,^54,55^ and when present, rates are extremely low.^49,56^ Although nociceptors do not synapse directly onto WDR neurons in the lamina V region,^57^ activity from these neurons may contribute indirectly to the increase in wind-up.

The lack of an increase in ongoing activity from WDR neurons following neuritis and vinblastine treatment is consistent with the minor nature of the insult. In particular, it is likely to reflect the absence of injury-induced discharge from Aβ-fiber neurons in both models,^3,33,48,53^ since many of these neurons synapse directly in lamina V,^57^ and ongoing activity from WDR neurons is reported to be directly driven by injury-induced activity from primary sensory neurons.^58^ In contrast, in models of traumatic nerve injury and chronic hind paw inflammation, where ongoing activity from WDR neurons is reported,^11,13–15^ ongoing activity from Aβ-fiber neurons is also a prominent feature.^52,54,55,59^

Although cutaneous mechanical hypersensitivity developed following both neuritis and vinblastine-induced axonal transport disruption, WDR neurons did not increase their response to direct mechanical stimulation of the receptive field. Instead, following vinblastine treatment, there was a notable decrease in the responsiveness of WDR neurons to noxious cutaneous mechanical stimulation (i.e. 60 g von Frey monofilament). This decrease may be due to altered neuronal connectivity or inhibition within the dorsal horn, leading to dampening of the nociceptive input. For example, activation of descending inhibitory pathways that act on dorsal horn neurons has been demonstrated following chronic inflammation of the hind paw.^60^ A similar central mechanism might also explain the reduction in the responses to innocuous mechanical stimulation of the receptive fields of WDR neurons at depths of ≥550 µm compared to those <550 µm. This finding also suggests that there are sub-populations of WDR neurons that respond differently to the treatment. However, the present data cannot rule out a peripheral mechanism, such as a decrease in the mechanical sensitivity of the peripheral terminals of nociceptors. Mechanically sensitive ion channel components are transported to the peripheral terminals by fast axonal transport,^61^ and therefore if transport is impeded, it may impact the mechanical sensitivity of nociceptors. Although neuritis also disrupts axonal transport, it occurs at a later time-point compared to vinblastine treatment,^33^ which might explain why a similar decrease in noxious mechanical sensitivity was not observed.

The increase in C-fiber stimulated c-Fos expression in superficial laminae in both the neuritis and vinblastine-treated groups compared to untreated suggests that these neurons, which were not examined electrophysiologically, were also sensitized; c-Fos is an indirect indicator of central sensitization, since its expression is driven by the activation of cAMP response element-binding protein (CREB), which mediates transcriptional changes that are associated with such mechanisms.^24^ Our finding is in line with previous studies that have examined activity-dependent c-Fos expression in nerve injury models.^25–27^ Although A-fiber–stimulated c-Fos expression was increased in all groups, which was most likely due to the activation Aβ- and Aδ-fiber nociceptors, there was no significant difference in expression between the experimental and untreated groups. This finding contrasts from reports in models of traumatic nerve injury,^25,62,63^ and a model of vincristine-induced neuropathy,^64^ and may reflect the minor nature of the injury.

Based on the peripheral generator theorem, the transient development of neuropathic pain behaviors and increased c-Fos expression in superficial laminae following vinblastine treatment seems incongruous with the reported lack of increased peripheral ongoing activity in this model.^33,53^ However, the decrease in substance P levels in the dorsal horn suggests that nociceptor transmission is altered. This decrease may be caused by the repeated activation of nociceptors (such as the activation of mechanically sensitive nociceptive axons at the treatment site during limb movements that stretch nerves^33,53^), which may cause the depletion of neuropeptides from central terminals.^65^ Alternatively, it may be the result of altered synthesis of substance P due to the reduced retrograde transport of trophic factors (e.g. nerve growth factor) from the peripheral terminals to the cell bodies; nerve growth factor is considered to modulate substance P production.^28,66,67^ The lack of a similar loss of substance P following neuritis might reflect the slower time course of axonal transport disruption.

The development of contralateral cutaneous hypersensitivities following neuritis and vinblastine treatment is consistent with a central mechanism. Similar contralateral cutaneous hypersensitivities have been reported following nerve injury,^16,68–71^ and chronic hind limb inflammation.^72,73^ Although contralateral dorsal horn neurons were not examined, previous studies have demonstrated increased excitability of WDR neurons on the contralateral side following nerve injury,^16,17,74^ which may underlie bilateral pain behaviors. Such contralateral changes in the dorsal horn may be driven by the activation of commissural interneurons (reviewed in Koltzenburg et al.^75^). However, a systemic mechanism involving the release of inflammatory factors from the neuritis site cannot be ruled out.

In summary, we have shown that both neuritis and vinblastine-induced axonal transport disruption, which are relatively minor nerve insults, cause functional changes to dorsal horn neurons. Whereas neuritis caused signs of increased excitability of deep WDR neurons, vinblastine treatment did not, which indicates mechanistic differences between models and a role for inflammation. It is unclear whether deep WDR neurons are involved in the development of cutaneous hypersensitivity. The reduction in their responses to mechanical stimulation following vinblastine treatment suggests that these neurons may not be part of the mechanism in this model. However, the sensitization of deep WDR neurons to cold stimuli, and therefore possible role in cold hypersensitivity, cannot be ruled out in either model. Since there was clear evidence for an increase in c-Fos expression in lamina I and II in both models, it is conceivable that these superficial neurons play a role in the underlying mechanisms. Based on the present and previous studies,^1,3,4^ an active neuritis is a possible explanation for neuropathic pain in those patients diagnosed with conditions such as non-specific arm or back pain, whiplash-associated disorder, fibromyalgia and complex regional pain syndrome where an obvious nerve injury may not be apparent on routine clinical testing. Data from our studies suggest that in this patient group, localized nerve inflammation may drive both peripheral and central mechanisms.

Axonal transport disruption is a major component of any nerve injury, whether the nerve is severed or inflamed. Locally treating nerves with vinblastine provides an alternative approach for examining the role of axonal transport disruption. With regards to animal welfare, it provides a refined, less severe model that could in part replace the use of frank nerve injury models. Importantly, local vinblastine treatment allows axonal transport disruption to be examined along intact axons, which may also be relevant for understanding the mechanisms of chemotherapy-induced neuropathy. While many laboratory studies have focused on traumatic nerve injury, axonal transport disruption along intact axons may in part underlie many of the chronic pain conditions that are seen in the clinic.

## Authors’ note

Data created during this research are openly available from the University of Sussex data archive at https://doi.org/10.25377/sussex.7048301.

## References

[1] Eliav E Herzberg U Ruda MA Bennett GJ. Neuropathic pain from an experimental neuritis of the rat sciatic nerve. Pain 1999; 83: 169–182. 10534588 10.1016/s0304-3959(99)00102-5

[2] Pulman KG Smith M Mengozzi M Ghezzi P Dilley A. The erythropoietin-derived peptide ARA290 reverses mechanical allodynia in the neuritis model. Neuroscience 2013; 233: 174–183. 23262243 10.1016/j.neuroscience.2012.12.022

[3] Bove GM Ransil BJ Lin HC Leem JG. Inflammation induces ectopic mechanical sensitivity in axons of nociceptors innervating deep tissues. J Neurophysiol 2003; 90: 1949–1955. 12724363 10.1152/jn.00175.2003

[4] Dilley A Lynn B Pang SJ. Pressure and stretch mechanosensitivity of peripheral nerve fibres following local inflammation of the nerve trunk. Pain 2005; 117: 462–472. 16154692 10.1016/j.pain.2005.08.018 PMC1402335

[5] Dilley A Bove GM. Resolution of inflammation-induced axonal mechanical sensitivity and conduction slowing in C-fiber nociceptors. J Pain 2008; 9: 185–192. 18309534 10.1016/j.jpain.2007.10.012

[6] Richards N Dilley A. Contribution of hyperpolarization-activated channels to heat hypersensitivity and ongoing activity in the neuritis model. Neuroscience 2015; 284: 87–98. 25290015 10.1016/j.neuroscience.2014.08.058

[7] Greening J Anantharaman K Young R Dilley A. Evidence for increased MRI signal intensity and morphological changes in the brachial plexus and median nerves of patients with chronic arm and neck pain following whiplash injury. J Orthopaed Sports Phys Ther 2018; 48: 523–532. 10.2519/jospt.2018.7875 29690828

[8] Dilley A Greening J Walker-Bone K Good C. Magnetic resonance imaging signal hyperintensity of neural tissues in diffuse chronic pain syndromes: a pilot study. Musc Nerve 2011; 44: 981–984. 10.1002/mus.22221 22102470

[9] Banic B Petersen-Felix S Andersen OK Radanov BP Villiger PM Arendt-Nielsen L Curatolo M. Evidence for spinal cord hypersensitivity in chronic pain after whiplash injury and in fibromyalgia. Pain 2004; 107: 7–15. 14715383 10.1016/j.pain.2003.05.001

[10] Stein C Millan MJ Herz A. Unilateral inflammation of the hindpaw in rats as a model of prolonged noxious stimulation: alterations in behavior and nociceptive thresholds. Pharmacol Biochem Behav 1988; 31: 445–451. 3244721 10.1016/0091-3057(88)90372-3

[11] Laird JM Bennett GJ. An electrophysiological study of dorsal horn neurons in the spinal cord of rats with an experimental peripheral neuropathy. J Neurophysiol 1993; 69: 2072–2085. 8394412 10.1152/jn.1993.69.6.2072

[12] Suzuki R Matthews EA Dickenson AH. Comparison of the effects of MK-801, ketamine and memantine on responses of spinal dorsal horn neurones in a rat model of mononeuropathy. Pain 2001; 91: 101–109. 11240082 10.1016/s0304-3959(00)00423-1

[13] Pertovaara A Kontinen VK Kalso EA. Chronic spinal nerve ligation induces changes in response characteristics of nociceptive spinal dorsal horn neurons and in their descending regulation originating in the periaqueductal gray in the rat. Exp Neurol 1997; 147: 428–436. 9344567 10.1006/exnr.1997.6555

[14] Chapman V Suzuki R Dickenson AH. Electrophysiological characterization of spinal neuronal response properties in anaesthetized rats after ligation of spinal nerves L5-L6. J Physiol 1998; 507: 881–894. 9508847 10.1111/j.1469-7793.1998.881bs.x PMC2230815

[15] Chu KL Faltynek CR Jarvis MF McGaraughty S. Increased WDR spontaneous activity and receptive field size in rats following a neuropathic or inflammatory injury: implications for mechanical sensitivity. Neurosci Lett 2004; 372: 123–126. 15531101 10.1016/j.neulet.2004.09.025

[16] Takaishi K Eisele JH Jr Carstens E. Behavioral and electrophysiological assessment of hyperalgesia and changes in dorsal horn responses following partial sciatic nerve ligation in rats. Pain 1996; 66: 297–306. 8880853 10.1016/0304-3959(96)03023-0

[17] Behbehani MM Dollberg-Stolik O. Partial sciatic nerve ligation results in an enlargement of the receptive field and enhancement of the response of dorsal horn neurons to noxious stimulation by an adenosine agonist. Pain 1994; 58: 421–428. 7838592 10.1016/0304-3959(94)90137-6

[18] Hylden JLK Nahin RL Traub RJ Dubner R. Expansion of receptive fields of spinal lamina 1 projection neurones in rats with unilateral adjuvant-induced inflammation: the contribution of dorsal horn mechanisms. Pain 1989; 37: 229–243. 2664665 10.1016/0304-3959(89)90135-8

[19] Li J Simone DA Larson AA. Windup leads to characteristics of central sensitization. Pain 1999; 79: 75–82. 9928779 10.1016/S0304-3959(98)00154-7

[20] Traub RJ. Spinal modulation of the induction of central sensitization. Brain Res 1997; 778: 34–42. 9462875 10.1016/s0006-8993(97)00946-3

[21] Herrero JF Cervero F. Changes in nociceptive reflex facilitation during carrageenan-induced arthritis. Brain Res 1996; 717: 62–68. 8738254 10.1016/0006-8993(95)01585-x

[22] Stanfa LC Sullivan AF Dickenson AH. Alterations in neuronal excitability and the potency of spinal mu, delta and kappa opioids after carrageenan-induced inflammation. Pain 1992; 50: 345–354. 1280804 10.1016/0304-3959(92)90040-I

[23] Hunt SP Pini A Evan G. Induction of c-fos–like protein in spinal cord neurons following sensory stimulation. Nature 1987; 328: 632–634. 3112583 10.1038/328632a0

[24] Latremoliere A Woolf CJ. Central sensitization: a generator of pain hypersensitivity by central neural plasticity. J Pain 2009; 10: 895–926. 19712899 10.1016/j.jpain.2009.06.012 PMC2750819

[25] Molander C Hongpaisan J Persson JK. Distribution of c-fos expressing dorsal horn neurons after electrical stimulation of low threshold sensory fibers in the chronically injured sciatic nerve. Brain Res 1994; 644: 74–82. 8032952 10.1016/0006-8993(94)90349-2

[26] Molander C Hongpaisan J Shortland P. Somatotopic redistribution of c-fos expressing neurons in the superficial dorsal horn after peripheral nerve injury. Neuroscience 1998; 84: 241–253. 9522378 10.1016/s0306-4522(97)00375-8

[27] Catheline G Le Guen S Honoré P Besson JM. Are there long-term changes in the basal or evoked Fos expression in the dorsal horn of the spinal cord of the mononeuropathic rat? Pain 1999; 80: 347–357. 10204748 10.1016/s0304-3959(98)00234-6

[28] Fitzgerald M Wall PD Goedert M Emson PC. Nerve growth factor counteracts the neurophysiological and neurochemical effects of chronic sciatic nerve section. Brain Res 1985; 332: 131–141. 2581648 10.1016/0006-8993(85)90396-8

[29] Garrison CJ Dougherty PM Carlton SM. Quantitative analysis of substance P and calcitonin gene-related peptide immunohistochemical staining in the dorsal horn of neuropathic MK-801-treated rats. Brain Res 1993; 607: 205–214. 7683239 10.1016/0006-8993(93)91508-p

[30] Villar MJ Wiesenfeld-Hallin Z Xu X-J Theodorsson E Emson PC Hökfelt T. Further studies on galanin-, substance P-, and CGRP-like immunoreactivities in primary sensory neurons and spinal cord: effects of dorsal rhizotomies and sciatic nerve lesions. Exp Neurol 1991; 112: 29–39. 1707368 10.1016/0014-4886(91)90111-o

[31] Abbadie C Brown JL Mantyh PW Basbaum AI. Spinal cord substance P receptor immunoreactivity increases in both inflammatory and nerve injury models of persistent pain. Neuroscience 1996; 70: 201–209. 8848125 10.1016/0306-4522(95)00343-h

[32] Galeazza MT Garry MG Yost HJ Strait KA Hargreaves KM Seybold VS. Plasticity in the synthesis and storage of substance P and calcitonin gene-related peptide in primary afferent neurons during peripheral inflammation. Neuroscience 1995; 66: 443–458. 7477885 10.1016/0306-4522(94)00545-g

[33] Dilley A Richards N Pulman KG Bove GM. Disruption of fast axonal transport in the rat induces behavioral changes consistent with neuropathic pain. J Pain 2013; 14: 1437–1449. 24035352 10.1016/j.jpain.2013.07.005

[34] Fitzgerald M Woolf CJ Gibson SJ Mallaburn PS. Alterations in the structure, function, and chemistry of C fibers following local application of vinblastine to the sciatic nerve of the rat. J Neurosci 1984; 4: 430–441. 6199484 10.1523/JNEUROSCI.04-02-00430.1984 PMC6564914

[35] Kashiba H Senba E Kawai Y Ueda Y Tohyama M. Axonal blockade induces the expression of vasoactive intestinal polypeptide and galanin in rat dorsal root ganglion neurons. Brain Res 1992; 577: 19–28. 1381649 10.1016/0006-8993(92)90532-e

[36] Katoh K Tohyama M Noguchi K Senba E. Axonal flow blockade induces alpha-CGRP mRNA expression in rat motoneurons. Brain Res 1992; 599: 153–157. 1283560 10.1016/0006-8993(92)90864-6

[37] Norris DA Weston WL Sams WM Jr. The effect of immunosuppressive and anti-inflammatory drugs on monocyte function in vitro. J Lab Clin Med 1977; 90: 569–580. 894108

[38] Dilley A Bove GM. Disruption of axoplasmic transport induces mechanical sensitivity in intact rat C-fibre nociceptor axons. J Physiol 2008; 586: 593–604. 18006580 10.1113/jphysiol.2007.144105 PMC2375581

[39] Dixon WJ. Efficient analysis of experimental observations. Annu Rev Pharmacol Toxicol 1980; 20: 441–462. 7387124 10.1146/annurev.pa.20.040180.002301

[40] Chaplan SR Bach FW Pogrel JW Chung JM Yaksh TL. Quantitative assessment of tactile allodynia in the rat paw. J Neurosci Methods 1994; 53: 55–63. 7990513 10.1016/0165-0270(94)90144-9

[41] Yoon C Wook YY Sik NH Ho KS Mo CJ. Behavioral signs of ongoing pain and cold allodynia in a rat model of neuropathic pain. Pain 1994; 59: 369–376. 7708411 10.1016/0304-3959(94)90023-X

[42] Dickenson AH Sullivan AF. Differential effects of excitatory amino acid antagonists on dorsal horn nociceptive neurones in the rat. Brain Res 1990; 506: 31–39. 1967963 10.1016/0006-8993(90)91195-m

[43] Xu XJ Zhang X Hokfelt T Wiesenfeld-Hallin Z. Plasticity in spinal nociception after peripheral nerve section: reduced effectiveness of the NMDA receptor antagonist MK-801 in blocking wind-up and central sensitization of the flexor reflex. Brain Res 1995; 670: 342–346. 7743203 10.1016/0006-8993(94)01360-t

[44] Woolf CJ. Central sensitization: implications for the diagnosis and treatment of pain. Pain 2011; 152: S2–S15. 20961685 10.1016/j.pain.2010.09.030 PMC3268359

[45] Gracely RH Lynch SA Bennett GJ. Painful neuropathy: altered central processing maintained dynamically by peripheral input. Pain 1992; 51: 175–194. 1484715 10.1016/0304-3959(92)90259-E

[46] Xie W Strong JA Meij JT Zhang JM Yu L. Neuropathic pain: early spontaneous afferent activity is the trigger. Pain 2005; 116: 243–256. 15964687 10.1016/j.pain.2005.04.017 PMC1343516

[47] Campbell JN Meyer RA. Mechanisms of neuropathic pain. Neuron 2006; 52: 77–92. 17015228 10.1016/j.neuron.2006.09.021 PMC1810425

[48] Bove GM Dilley A. The conundrum of sensitization when recording from nociceptors. J Neurosci Methods 2010; 188: 213–218. 20171245 10.1016/j.jneumeth.2010.02.010 PMC2854223

[49] Djouhri L Koutsikou S Fang X McMullan S Lawson SN. Spontaneous pain, both neuropathic and inflammatory, is related to frequency of spontaneous firing in intact C-fiber nociceptors. J Neurosci 2006; 26: 1281–1292. 16436616 10.1523/JNEUROSCI.3388-05.2006 PMC6674571

[50] Kocher L Anton F Reeh PW Handwerker HO. The effect of carrageenan-induced inflammation on the sensitivity of unmyelinated skin nociceptors in the rat. Pain 1987; 29: 363–373. 3614971 10.1016/0304-3959(87)90051-0

[51] Kelly S Dunham JP Murray F Read S Donaldson LF Lawson SN. Spontaneous firing in C-fibers and increased mechanical sensitivity in A-fibers of knee joint-associated mechanoreceptive primary afferent neurones during MIA-induced osteoarthritis in the rat. Osteoarthritis Cartilage 2012; 20: 305–313. 22285737 10.1016/j.joca.2012.01.002

[52] Xiao WH Bennett GJ. Persistent low-frequency spontaneous discharge in A-fiber and C-fiber primary afferent neurons during an inflammatory pain condition. Anesthesiology 2007; 107: 813–821. 18073557 10.1097/01.anes.0000286983.33184.9c

[53] Satkeviciute I Goodwin G Bove GM Dilley A. The time course of ongoing activity during neuritis and following axonal transport disruption. J Neurophysiol 2018; 119: 1993–2000. 29465329 10.1152/jn.00882.2017 PMC7938769

[54] Boucher TJ Okuse K Bennett DLH Munson JB Wood JN McMahon SB. Potent analgesic effects of GDNF in neuropathic pain states. Science 2000; 290: 124–127. 11021795 10.1126/science.290.5489.124

[55] Liu X Eschenfelder S Blenk KH Janig W Habler H. Spontaneous activity of axotomized afferent neurons after L5 spinal nerve injury in rats. Pain 2000; 84: 309–318. 10666536 10.1016/s0304-3959(99)00211-0

[56] Wu G Ringkamp M Hartke TV Murinson BB Campbell JN Griffin JW Meyer RA. Early onset of spontaneous activity in uninjured C-fiber nociceptors after injury to neighboring nerve fibers. J Neurosci 2001; 21: RC140 11306646 10.1523/JNEUROSCI.21-08-j0002.2001 PMC6762537

[57] Todd AJ. Neuronal circuitry for pain processing in the dorsal horn. Nat Rev Neurosci 2010; 11: 823–836. DOI: 10.1038/nrn2947. 21068766 10.1038/nrn2947 PMC3277941

[58] Pitcher GM Henry JL. Governing role of primary afferent drive in increased excitation of spinal nociceptive neurons in a model of sciatic neuropathy. Exp Neurol 2008; 214: 219–228. 18773893 10.1016/j.expneurol.2008.08.003 PMC5132624

[59] Kajander KC Wakisaka S Bennett GJ. Spontaneous discharge originates in the dorsal root ganglion at the onset of a painful peripheral neuropathy in the rat. Neurosci Lett 1992; 138: 225–228. 1319012 10.1016/0304-3940(92)90920-3

[60] Green GM Lyons L Dickenson AH. Alpha2-adrenoceptor antagonists enhance responses of dorsal horn neurones to formalin induced inflammation. Eur J Pharmacol 1998; 347: 201–204. 9653882 10.1016/s0014-2999(98)00217-9

[61] Koschorke GM Meyer RA Campbell JN. Cellular components necessary for mechanoelectrical transduction are conveyed to primary afferent terminals by fast axonal transport. Brain Res 1994; 641: 99–104. 8019856 10.1016/0006-8993(94)91820-1

[62] Bester H Beggs S Woolf CJ. Changes in tactile stimuli-induced behavior and c-Fos expression in the superficial dorsal horn and in parabrachial nuclei after sciatic nerve crush. J Comp Neurol 2000; 428: 45–61. 11058224 10.1002/1096-9861(20001204)428:1<45::aid-cne5>3.0.co;2-a

[63] Kosai K Tateyama S Ikeda T Uno T Nishimori T Takasaki M. MK-801 reduces non-noxious stimulus-evoked Fos-like immunoreactivity in the spinal cord of rats with chronic constriction nerve injury. Brain Res 2001; 910: 12–18. 11489249 10.1016/s0006-8993(01)02715-9

[64] Thibault K Rivals I M’Dahoma S Dubacq S Pezet S Calvino B. Structural and molecular alterations of primary afferent fibres in the spinal dorsal horn in vincristine-induced neuropathy in rat. J Mol Neurosci 2013; 51: 880–892. 23975629 10.1007/s12031-013-0095-4

[65] Klein CM Coggeshall RE Carlton SM Westlund KN Sorkin LS. Changes in calcitonin gene-related peptide immunoreactivity in the rat dorsal horn following electrical stimulation of the sciatic nerve. Neurosci Lett 1990; 115: 149–154. 2234494 10.1016/0304-3940(90)90446-g

[66] Nielsch U Bisby MA Keen P. Effect of cutting or crushing the rat sciatic nerve on synthesis of substance P by isolated L5 dorsal root ganglia. Neuropeptides 1987; 10: 137–145. 2446191 10.1016/0143-4179(87)90015-1

[67] Goedert M Stoeckel K Otten U. Biological importance of the retrograde axonal transport of nerve growth factor in sensory neurons. Proc Natl Acad Sci USA 1981; 78: 5895–5898. 6170988 10.1073/pnas.78.9.5895 PMC348900

[68] Seltzer Z Dubner R Shir Y. A novel behavioral model of neuropathic pain disorders produced in rats by partial sciatic nerve injury. Pain 1990; 43: 205–218. 1982347 10.1016/0304-3959(90)91074-S

[69] Kim SH Chung JM. An experimental model for peripheral neuropathy produced by segmental spinal nerve ligation in the rat. Pain 1992; 50: 355–363. 1333581 10.1016/0304-3959(92)90041-9

[70] Tabo E Jinks SL Eisele JH Carstens E. Behavioral manifestations of neuropathic pain and mechanical allodynia, and changes in spinal dorsal horn neurons, following L4-L6 dorsal root constriction in rats. Pain 1999; 80: 503–520. 10342412 10.1016/S0304-3959(98)00243-7

[71] Arguis MJ Perez J Martinez G Ubre M Gomar C. Contralateral neuropathic pain following a surgical model of unilateral nerve injury in rats. Reg Anesth Pain Med 2008; 33: 211–216. 18433671 10.1016/j.rapm.2007.12.003

[72] Radhakrishnan R Moore SA Sluka KA. Unilateral carrageenan injection into muscle or joint induces chronic bilateral hyperalgesia in rats. Pain 2003; 104: 567–577. 12927629 10.1016/s0304-3959(03)00114-3 PMC2732018

[73] Gao YJ Xu ZZ Liu YC Wen YR Decosterd I Ji RR. The c-Jun *N*-terminal kinase 1 (JNK1) in spinal astrocytes is required for the maintenance of bilateral mechanical allodynia under a persistent inflammatory pain condition. Pain 2010; 148: 309–319. 20022176 10.1016/j.pain.2009.11.017 PMC2814908

[74] Pitcher GM Henry JL. Cellular mechanisms of hyperalgesia and spontaneous pain in a spinalized rat model of peripheral neuropathy: changes in myelinated afferent inputs implicated. Eur J Neurosci 2000; 12: 2006–2020. 10886340 10.1046/j.1460-9568.2000.00087.x

[75] Koltzenburg M Wall PD McMahon SB. Does the right side know what the left is doing? Trends Neurosci 1999; 22: 122–127. 10199637 10.1016/s0166-2236(98)01302-2

